# Biomimetics for Sustainable Developments—A Literature Overview of Trends

**DOI:** 10.3390/biomimetics8030304

**Published:** 2023-07-11

**Authors:** Anne-Sophie Jatsch, Shoshanah Jacobs, Kirsten Wommer, Kristina Wanieck

**Affiliations:** 1 Faculty of Applied Informatics, Deggendorf Institute of Technology (DIT), Teaching Area Biomimetics and Innovation, Grafenauer Str. 22, 94078 Freyung, Germanykirsten.wommer@th-deg.de (K.W.); 2 Department of Integrative Biology, College of Biological Sciences, University of Guelph, Guelph, ON N1G 2W1, Canada; sjacob04@uoguelph.ca

**Keywords:** sustainability, bio-inspired design, innovation, sustainable development goals

## Abstract

Biomimetics holds the promise to contribute to sustainability in several ways. However, it remains unclear how the two broad concepts and research fields are connected. This article presents a literature overview on biomimetic sustainable developments and research. It is shown that there is an increasing trend in publications dealing with various topics and that the research takes place worldwide. The biological models studied in biomimetic sustainable developments are mostly sub-elements of biological systems on a molecular level and lead to eco-friendly, resource and energy-efficient applications. This article indicates that biomimetics is further integrating sustainability to contribute to real problems in this context.

## 1. Introduction

Biomimetics has become an important research and development paradigm of the past decade, mostly in technology-oriented fields but also with impacts on society and the economy [1]. Though promising for innovation and sustainable design, one of its big challenges is addressing real problems that lead to commercial products in the market [2]. With an urgent need to ramp up sustainable design within biomimetic research, we investigate how biomimetics has thus far contributed to sustainable technological design.

With the climate crisis, food insecurity and diminishing access to health care, the challenges of this current decade have become increasingly urgent as presented in the Sustainable Development Goals (SDG) [3]. Biomimetics holds the promise to potentially contribute to sustainable design [4], and to do so, clear methodologies need to be developed on how to respect this topic in biomimetic developments. Several previous projects focused on such connection [5,6,7], and the potential is well-known [8,9].

The international standard ISO 18458 [4] speaks of a “biomimetic promise” to sustainability but that biomimetics is not sustainable per se, as already coined by von Gleich et al. 2010 [10]. Researchers are increasingly paying attention to the contribution of biomimetics to the environmental sustainability of products and processes and are looking for ways to reproducibly measure and evaluate this contribution [6,7,11,12,13,14,15]. Previous research shows that it is possible to assess sustainability in bio-inspired developments in various contexts. One example is a biomimetic lightweight construction that is comparable to traditional developments regarding its ecological sustainability [12]. It was also shown that a façade painting, inspired by the lotus plant, was a resource-efficient product that was ecologically more favorable than a compared conventional product [13]. In the context of architecture, it was shown that a bio-based indoor panel can compete with conventional products, even though the research on the biomimetic product and the assessment methodology was still ongoing [14]. For all those sustainability assessments, various tools were used, and ecological, economic and social aspects of sustainability were analyzed, which shows the challenge and complexity of bringing biomimetics and sustainability together.

The practice of biomimetics so far lacks a clear connection to sustainability for several reasons. (a) Sustainability needs a clear definition in the context of biomimetics. Previous research shows that several connections exist [11,13,15,16,17], ranging from how to understand sustainability to how to train people to engage in the topic. (b) Sustainability in biological models needs to be defined. It encompasses topics like energy efficiency, material efficiency, low temperature production, usage of less harmful substances and optimized processes amongst others. The complexity of organisms and key concepts in this context have also been discussed [18]. (c) Biological principles that abstract functions and address sustainability need to be defined. One such approach is the so called Life’s principles [19,20]. These are common strategies and principles in biological systems to achieve certain functions. Those functions can also be defined in a technological realm. Such a list of principles enables knowledge transfer from technology to biology and vice versa. In this specific example, a focus is set on sustainable designs when applying those principles. However, it still needs to be investigated and communicated how such principles can be integrated into the biomimetic design process and, particularly, used in problem-solving.

Like sustainability, biomimetics is a very broad field [21] with even broader application. Specifically, at the intersection between biomimetics and sustainability, we observe synergies that range from biomimetics as a method for sustainable innovation and products to new sustainability strategies of businesses [16,22].

To better understand the existing practices in sustainable biomimetics, we conduct an analysis of the scientific literature, performed much in the same way as Jacobs et al. (2014) who characterized the current state of biomimetics practice [23]. This work can be considered an update with a focus on sustainability. Additionally, this research aims to figure out the connection between biomimetics and sustainability and how biomimetics is understood and used in this context The aim of this manuscript is to give an overview of the existing research, to present insights, to indicate trends, and to encourage future research. With this manuscript, we want to address the following questions:

How is sustainability already integrated into biomimetics research?
Are there differences among the broad terminology of bio-inspired design?
Which are the most prominent research areas?
Which countries publish the most research in the field?
To address these questions, we conduct a literature review.

## 2. Materials and Methods

### 2.1. Literature Review

The Web of Science (WoS) search engine was used for the analysis. WoS allows parallel and interdisciplinary searching of an extensive interdisciplinary database that is publisher-independent, is global and contains over 21,100 peer-reviewed scientific journals, including open access journals in over 250 scientific fields. Conference proceedings and books are also included. The results presented below are as of 30 June 2021.

Six different keywords of biomimetics were used for the literature search (“biomimetic*”, “bio*-inspir*”, “bioinspir*”, “biomimic*”, “natur*-inspir*”, “bionic*”), and each was combined with “sustainab*” for the topic sustainability, while “*” represents variable endings of the words. When comparisons of the terms were made with the data from Jacobs et al. 2014 [23], these six words were combined into five categories: “biomimetic*”, “bioinspir*/bio-inspir*”, “biomimic*”, “bionic*” and “other”. Due to the similarity of the search terms, 239 of the 1381 publications were found more than once. These were not considered for further analysis. This left 1142 publications for evaluation. Most publications that were found more than once had already been identified with the search term “biomimetic*”. The results for “biomimetic*” were listed first, and any publication that had already been identified there was eliminated from the other search terms.

From the search results, title, year of publication, journal/conference, authors, country of first author, abstract, and keywords were extracted and recorded.

For an article to be included, both the biomimetics (or associated) term and the sustainability (or associated) term needed to be used within the text. The complete collection of articles was analyzed for the following variables:

1. Search terms that yielded the article
2. Title of the article
3. Publishing year
4. Journal/conference
5. Authors
6. Country
7. Full text/Abstract
8. Key points of the abstract
9. Target group
10. Research Area
11. Type of sustainability
12. Biological model

The classification was performed by a staff member with education in biology and verified by the co-authors of this article who have both biology and engineering training. The database was then analyzed for thematic context of the publications and for comparison with the analysis of the BioM Innovation Database published by Jacobs et al. in 2014 [23].

### 2.2. Data Analysis

**Figure 1:**
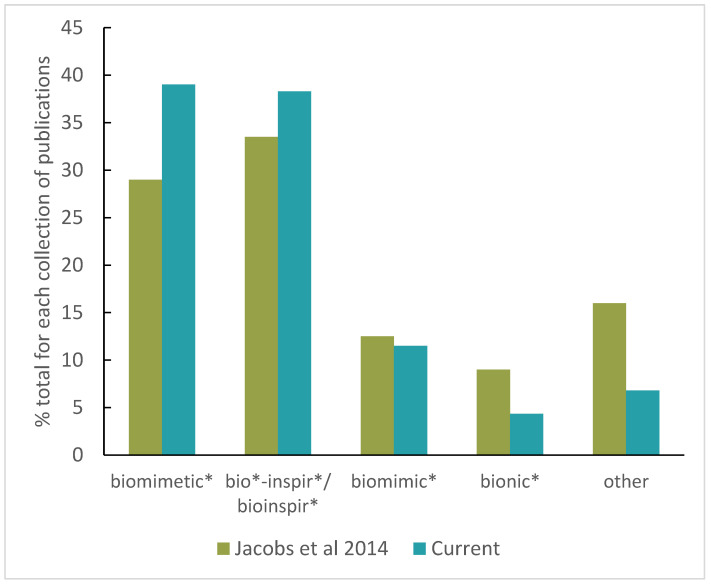
Proportion of publications based on different terms (*n* = 1381). Four different terms for biomimetics were searched in combination with “sustainab*”. The term “biomimetic*” had the most returns (*n* = 539; 39%), while the term “bionic*” had the fewest. Comparison with the distribution of terms from Jacobs et al. 2014 [23] (using only those technologies identified as being actually biomimetic; 1912–2012) reveals no significant change in the frequency of term use (X2 = 0.35, df = 1, *p* = 0.55).

We compared our data set with that of Jacobs et al. 2014 [23] to determine whether there was a difference in the distribution of search terms with sustainable application using a contingency table of analysis. This analysis allowed for the comparison of a frequency distribution across multiple categories. The results of the frequency distribution are presented in Figure 1 for easy comparison.

**Figure 2:**
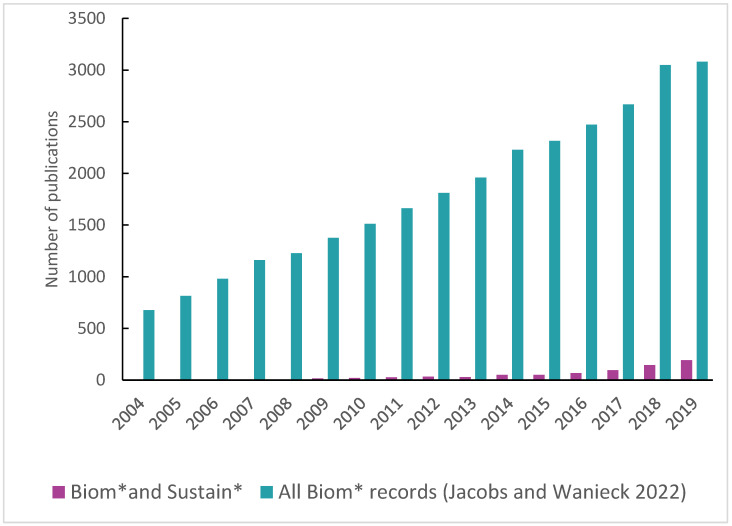
Number of publications on the topic of sustainability and biomimetics per year with all records of biomimetics for comparison. Shown is the number of publications per year since 2004 until 2019 (”biomimetic sustainable design” *n* = 1142 in purple; Biom* from Jacobs and Wanieck 2022 *n* = 29,161 in teal) [21].

**Figure 3:**
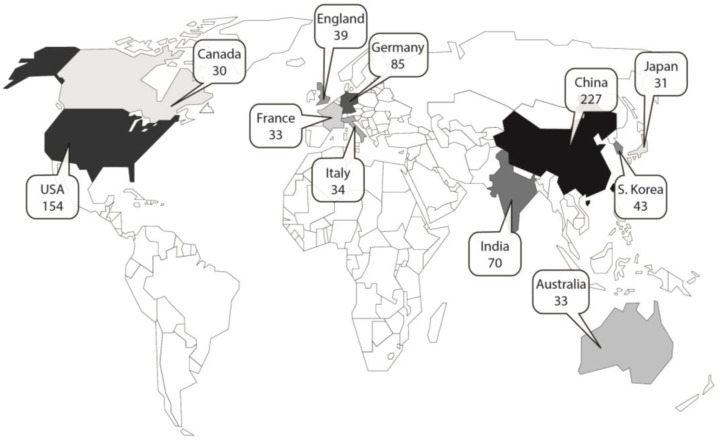
Number of publications on “Biomimetic sustainable design” (*n* = 1142) per country in the years 2004–2021. Shown is an overview of the number of published publications per country in which the first authors were researching at the time of publication. Only those countries from which at least five papers originate are shown.

**Figure 4:**
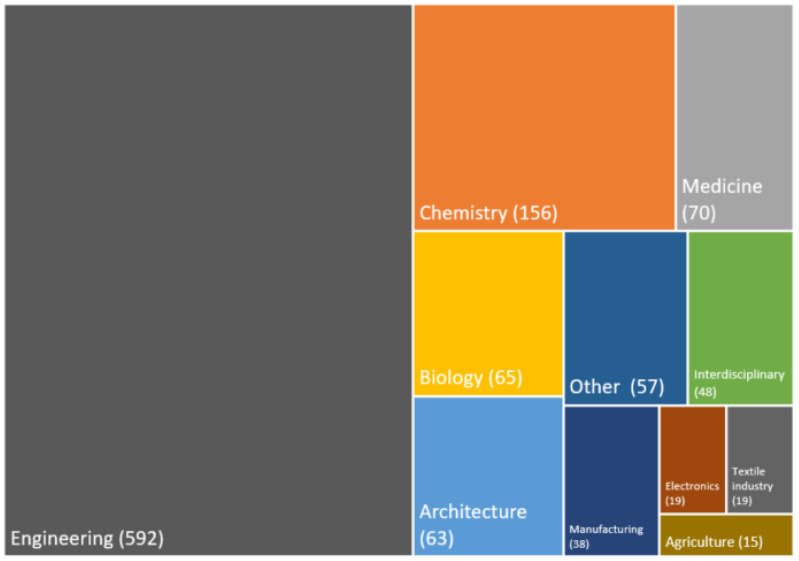
Discipline of application of the publications on biomimetic sustainable design (*n* = 1142).

**Figure 5:**
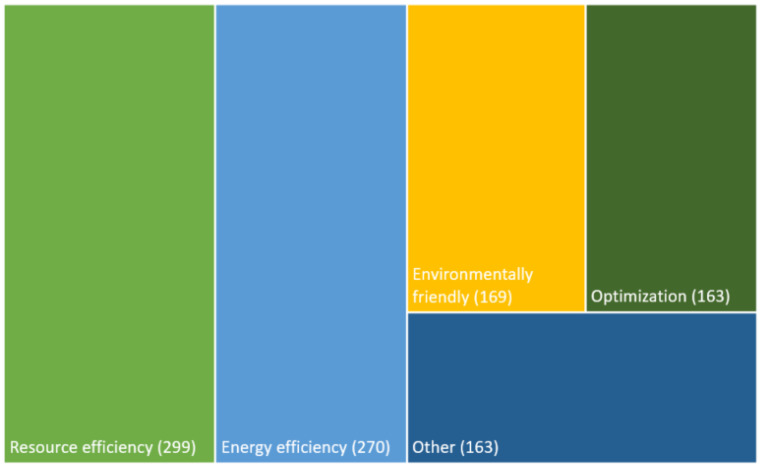
Classification of publications (*n* = 875) into different sustainability aspects.

**Figure 6:**
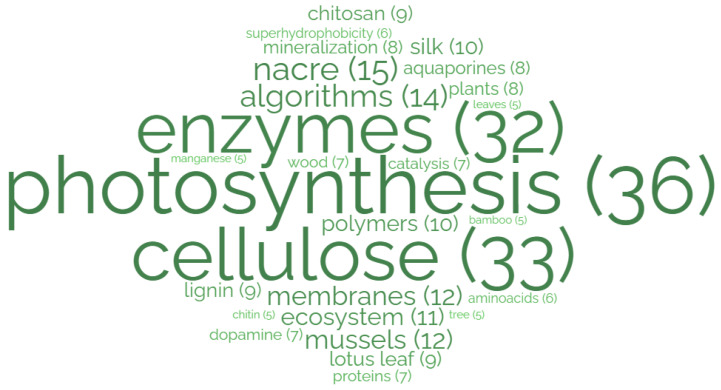
Biological models, sub-systems, principles, structures or organisms found in at least five publications. Photosynthesis serves most frequently as a model in the published research (*n* = 36).

We present the trends in the total number of articles published on the topic of biomimetics as presented on WoS from Jacobs and Wanieck 2022 and add those included in our review here that focused on sustainability [21]. We present these data in Figure 2 for comparison of the trends since 2004. Our geographical analysis focuses on the leading countries and is presented on a world map in Figure 3. We present the distribution of disciplines engaged in sustainable biomimetic research in a mosaic plot in Figure 4 to identify the dominant research areas. Tables 1 and 2 list the number of types of studies and the publications in which most of the research dissemination occurs. We identified the sustainability focus for each of the articles and present the distribution in a mosaic plot in Figure 5. We present the frequency of biological models in a word cloud for ease of visualization in Figure 6. The biological models or sub-systems were mentioned in the identified literature.

To provide more insight into the research field, the literature analyses was, in parts, repeated and extended in June 2023. The WoS database was searched again for the terms “biomimetics” and “biomimetics and sustainab*”. The results were analyzed for research area, country of origin as well as the Top 50 most cited papers for both search terms. We used the metric of most cited literature to identify the research areas that attract most attention.

### 2.3. Limitations of the Study

WoS was updated in September 2021. It is possible that WoS has since used additional databases not previously used; therefore, repeating the search could yield additional results. In many cases, the variables 9 and 10, the target group and the research area could not be clearly defined since the studies often overlapped in fields and did not clearly define an addressed target group. Depending on the background of a person, these variables can be understood in a different manner. Nevertheless, this extensive study provides a good overview about the state-of-the-art in sustainable applied biomimetics.

For the comparison of the literature on “biomimetics” and “biomimetics and sustainab*”, the data were analyzed as given by WoS (16 June 2023). It was not analyzed whether the overall resulting papers (*n* = 24.178) were correctly identified by WoS as “biomimetic” or “sustainable” by definition. This might lead to false-positive counts as observed, for instance, in Table S2 (Supplementary Materials) line 8. These search results were, therefore, analyzed to give general insight into the two research fields and to compare them rather than giving detailed insights as for the main data set and results shown in this manuscript.

The research areas given by WoS are specific categories, which might differ slightly in terminology between each search record analyzed with the system. Therefore, we refer to these areas as general trends and areas rather than making a clear distinction and drawing respective conclusions.

## 3. Results

The literature searches with the different combinations of terms returned a total of 1381 results for the keyword searches in the context of biomimetics and sustainability between the years 2004 and 2021 (Figure 1). Most results were found with the combination “sustainab*” and “biomimetic*” (*n* = 539), demonstrating that the term “biomimetics” is most frequently used for learning from nature in the context of sustainability, a pattern that has been consistent and also shown in the BioM innovation database [23].

The alternative terms for learning from nature each yielded fewer hits but collectively yielded more hits overall (*n* = 842). This illustrates that the research field of biomimetics is scattered and anchored in a variety of disciplines, which leads to the large number of different terms, making research and a clear classification more difficult. When compared with the distribution of terms used in Jacobs et al. (2014) using a contingency table analysis [23], we found no significant difference (X2 = 0.35, df = 1, *p* = 0.55). This demonstrates that the sustainability application of biomimetics is using the same distribution of biomimetics-related terms as the general field of biomimetics as analyzed 10 years ago.

The merged number of publications over all search terms was used, as it was not distinguished whether the terms used in the publications were used correctly with reference to the ISO definition of biomimetics [4]. In the following, this set of publications (*n* = 1142) will be referred to as publications in the context of “biomimetic sustainable design”.

The absolute number of biomimetics articles with a focus on sustainability remains low compared to the increasing number of biomimetics articles published each year (Figure 2). However, the number of publications with a focus on sustainability has been steadily increasing since 2016 (Figure 2). This suggests that there is increased interest in sustainable biomimetic production and that biomimetics is becoming more established in the research landscape, as the potential of nature as a role model and solution approach for technical problems is increasingly recognized.

A search in WoS in February 2020 showed that, in 2016, approximately 2500 publications were published with the search term “biomimetic”. In 2019, there were over 3000 publications [21]. For 2020, 3700 publications are currently displayed (retrieved 20/05/2022), indicating that publications on the topic of biomimetics and sustainability accounted for 7% (255 of 3700 publications) of biomimetics publications in 2020, 6% in 2019 (193 of 3081 publications) and 3% in 2016 (68 of 2472 publications).

In the international context, China (*n* = 227), USA (*n* = 154) and Germany (*n* = 85) have published the most research papers in the field of biomimetic sustainable design. (Figure 3).

A comparison of “biomimetics” and “biomimetics and sustainab*” literature shows that the top three publishing countries for these topics are the same as shown in Figure 3, i.e., China, USA and Germany. When the top 10 countries are compared for all three categories, nine nations are present in a slightly different order: China, USA, Germany, England/UK, South Korea, Japan, Italy, India and Australia. The tenth nation is France (biomimetics; biomimetic sustainable design) or Canada (biomimetics and sustainab*).

Twenty different application references of sustainable biomimetic research were identified (Figure 4). Most publications dealt with technology and engineering in general (*n* = 585). A total of 156 publications dealt with chemistry, 70 with medicine, 65 with biology, and 63 with architecture. Four publications could not be assigned to a specific category, and these were assigned to the ‘others’ category.

In architecture, biomimetics is used to design energy-efficient or smart buildings, e.g., biology often describes potential role models that can be used to solve engineering problems. Chemical reactions that are inspired by nature, for example, that mimic photosynthesis, are often presented in publications, optimized, or sustainable replacement components are sought. The topics that were counted into the field of medicine were predominantly about prostheses, like the human body parts or creating skin-like textures.

In addition to the thematic classification, the publications were also analyzed by type (Table 1). The publications predominantly present experimental results or reviews of existing research. In addition, case studies were conducted, or applications were presented that were developed and tested. Algorithms, procedural patterns, methods as well as prototypes were presented.

The information presented in the abstracts was used to assess whether the research publications (*n* = 1142) dealt with the topic of biomimetics and sustainability. This included the understanding of biomimetics according to ISO 18458 and the focus on *ecological* sustainability to narrow down the different dimensions of this broad topic [12,17].

A total of 875 publications met the criteria and were published in a total of 434 different journals (Table 2). Ten different journals published 152 papers in the context of biomimetic sustainable design. The rest of the identified papers (*n* = 723) were published in 424 different journals with an average of 1.7 papers per journal over the period 2004–2021 (Table 2). The names of the journals indicate the focus in current research topics related to sustainability and biomimetics. The focus here is on chemistry, materials, engineering, nanotechnologies and sustainability in general. The listed journals are of high reputation as measured with traditional metrics including impact factors. This demonstrates a continued appetite for biomimetics research that includes sustainability. The high number of different journals shows how scattered the field of biomimetics is, and this fact seems to be intensified in the connection to the equally broad topic of sustainability. To specify this research field, it is important to identify clusters in which research can take place so that the findings can lead to a clear contribution of biomimetics to the challenging issues of sustainability.

The 875 publications were classified into sub-areas of sustainability. One paper could be assigned to more than one topic; therefore, the total of publications across all focal areas is higher than the number of papers analyzed (*n*_publications_ = 875, *n*_classifications_ = 1064). Figure 5 shows the five most frequently identified sustainability aspects.

More than one third of the publications (299 of 875 papers = 34%) dealt with resource efficiency, e.g., [24]. Energy efficiency was addressed in 270 publications (~31%), e.g., [25]. A total of 169 publications dealt with environmental friendliness (~19%), e.g., [26]. Optimizations for increased sustainability were presented in 163 publications (~19%), e.g., [27]. Sustainability was addressed in general terms in 153 publications (~17%), e.g., [28]. In three other publications, tools/approaches for increased sustainability, for example, of products, were presented, such as algorithms. For seven publications, no specific assignment could be made based on the abstract.

Each biomimetic project involves, by definition, a biological model that is analyzed and abstracted [4]. The distribution of biological models is not without bias. Jacobs et al. 2014 found a strong bias towards the vertebrates with most of the described models focused on tissues and organ levels of biological organization [23]. In our sustainability filtered analysis, we find that ‘photosynthesis’ is the most often presented model, where designers are attempting to recreate the generation of energy from sunlight (Figure 6).

Interestingly, when compared with the distribution of models described in Jacobs et al. 2014, the overall biological level of organization is dropping towards the smaller levels with a strong emphasis on molecules [23]. This could be explained by either an increased accuracy in describing models within the literature or, more likely, a demonstration of the relevant levels of organization for successful model inspiration and transfer to sustainability technology.

To better understand the complexity and development of research in the field, the following tables enable a comparison between the literature on “biomimetics” and “biomimetics and sustainab*”. For “biomimetics”, a total of 23.735 papers were found on WoS (all databases, 1945–2023, 16 June 2023). For “biomimetics and sustainab*”, a total of 443 results were found. Table 3 summarizes the respective research areas.

As shown in Table 3, the research areas in biomimetics and biomimetics linked to sustainability are quite similar. The comparison of the two fields shows that biomimetics encompasses a wide variety of research areas both regarding the research itself and also potential fields of application. There are traditional disciplines involved, like biology, chemistry, physics, polymer sciences or material sciences. For both fields, the focus is on engineering and material science. The natural sciences are most represented through chemistry and physics followed by biotechnology/microbiology and then biochemistry/molecular biology. There are 17 research areas that can be found in both fields of which the first two research areas are the same in both research fields, i.e., engineering and material science. The eight specific areas for biomimetics are physiology, mathematics, automation control systems, anatomy morphology, neuroscience/neurology, mathematical computational biology, zoology and life sciences/biomedicine/others. The eight specific topics of biomimetics and sustainability are environmental sciences/ecology, energy fuels, business economics, education/educational research, electrochemistry, plant sciences, agriculture and construction building technology.

To analyze the research areas that are most recognized, the research areas of the top 50 most cited papers are given in Table 4.

Table 4 shows that the top 50 most cited papers from both biomimetics and biomimetics and sustainability cover disciplinary research areas, like physics, chemistry, material sciences, engineering, nanotechnology, computer science or biology with subfields. For biomimetics linked to sustainability, multidisciplinary sciences or energy fuels are also represented. In addition, a focus on multidisciplinary areas is indicated. Compared to Table 3, the most recognized publications are from research areas similar to the ones covered by the research in general.

To give more insight into the specific topics covered, Tables S1 and S2 list the top 20 most cited papers for the two fields as supplementary data. In addition, the type of article is given. Compared to Table 1, reviews are often recognized and cited.

The top 20 most cited papers of “biomimetics*” were published during the years 2003–2016. The most cited one has an average of 1029 citations per year. The lowest ranked paper is from 2011 and has an average of 118 citations per year. In comparison, the top 20 most cited papers of “biomimetics and sustainab*” were published during the years 2007–2020. The most cited one has an average of 307 citations per year. The lowest ranked paper is from 2019 and has an average of 37 citations per year. For biomimetics and sustainab*, there are more reviews in the top 20 list than for biomimetics in general.

## 4. Discussion

Regarding terminology, the literature review shows the diversity of biomimetics, its connectedness to various other disciplines as well as an inconsistent use of different terms. The use and understanding of different terms become even more important in the context of sustainability. Landrum and Mead (2022) give an overview of bio-inspired approaches and position them in a sustainability spectrum [17]. In their comparison, biomimicry is classified with a strong to very strong sustainability, while biomimetics is more intermediate, indicating that sustainability is not a priority in biomimetic design, even though the potential is there. Other studies support this classification [5,18]. In our study, we could show that biomimetics is the most used term in the scientific literature on biomimetic sustainable design (Figure 1). This raises the question of whether biomimetics will incorporate a focus on sustainable design in the future or whether the two approaches share more overlap than assumed so far.

The publications found in the context of biomimetic sustainable design during 2004–2021 accounted for up to 7% of the overall publications in biomimetics (Figure 2). We consider this a good starting point towards more sustainability-focused biomimetic research. As the interpretations of “biomimetics” and “sustainable” vary, it is expected that there is more research already going on that is not declared as biomimetic sustainable design regarding terminology and keywords. It is expected that the theory of biomimetics is developing, and therefore, its contribution to sustainability research will increase.

As a high variety of journals published papers regarding the topic biomimetics in combination with sustainability (Table 2), this shows that there is an increasing interest from many perspectives on biomimetics and that biomimetics can contribute to various research. There is hope that this trend will increase, as biomimetic research has just scratched the surface of biological inspiration [29].

Interestingly, regarding the biological models used in biomimetic sustainable design, we could observe a shift to molecular scale (Figure 6). This is in line with the development strands of biomimetics described by von Gleich et al., leading to nano biomimetics and respective developments [10]. Additionally, Vincent et al. 2006 indicated that there is a general difference between engineering problem-solving and biological strategies to solve problems in terms of scale [30]. The products analyzed in the BioM innovation database focus more on ecosystem bio-inspiration. It will be interesting to observe how biological models can be used on those different levels of adaptation, complexity and scale for future biomimetic sustainable design. Also, the models mentioned in Figure 6 needed to be analyzed in more detail, as it is not distinguished how sustainable, efficient or useful those models are or might be.

The national contribution (Figure 3) shows that China (*n* = 227), North America (*n* = 184) and Europe (*n* = 191) are the leading countries regarding the overall publications on the topic of biomimetics and sustainability. These three G20 members are the top three greenhouse gas emitters on an absolute basis [31]. It will be interesting to observe how the research topics, the trend of rising publications and the contribution of the research to measurable effects in solving problems will develop.

Regarding the various research topics (Figure 4) and the types of publications (Table 1), it seems that biomimetic sustainable design is mostly present in fundamental and applied research fields and that there is still a gap towards industrial application [32]. This was already shown with the BioM innovation database [23], as most cases analyzed were in development. Yet, biomimetics needs to solve real problems that will lead to real products in the market [2]. This is specifically true in the context of sustainability and climate change solutions, as new technologies are needed. The UN’s SDGs are already present in the literature review shown in the main topics. It will be interesting to observe whether biomimetics contributes to optimizing existing traditional technologies or if it will lead to true innovative solutions based on the principles that biological systems use. Table 1 lists reviews as the second most frequent type of publication. Also, the top 20 list of biomimetic and sustainab* literature includes more reviews than biomimetics. We did not analyze these reviews yet, but biomimetics linked to sustainability seems to provide knowledge of specific topical aspects of the field via reviews. It might also be that this connection is complex and needs broader explanation. Additionally, the field is still developing, and future research is expected to lead to more original research articles.

The eight specific research areas of biomimetics and sustainability are environmental sciences/ecology, energy fuels, business economics, education/educational research, electrochemistry, plant sciences, agriculture and construction building technology. This finding shows that the connection to sustainability offers additional specific fields of application. Also, the educational aspect seems to be even more important. In previous papers, we analyzed the educational pipeline of biomimetics and what is needed to foster knowledge transfer [33,34]. Once sustainability becomes part of biomimetics research, education and raising awareness as well as detailed explanations become more important. Also, the research in connection with sustainability becomes broader and more diverse as shown with areas like business economics or agriculture. Existing biomimetics research could further address sustainability to strengthen this research. We are sure that biological systems hold much more information that can be valuable for future research in the field.

Using the citation frequency as a metric to identify research areas that are highly recognized did not yield specific insights except that biomimetics linked to sustainability seems to be more multidisciplinary.

## 5. Conclusions

The literature overview shows that specific topics in the context of biomimetics and sustainability, like studying photosynthesis for new technologies, can have a great impact in fundamental and applied research and in solving current real problems. Therefore, it is necessary to have a clear picture of what biomimetic sustainable design can include and in which direction research currently takes place. The presented data are a first insight into the field, and future research will help draw the picture. Additionally, the analysis presented here can help identify funding opportunities, which is a known challenge in biomimetics [21].

We could name “biomimetics and sustainability” a next development strand of biomimetics. As observed in the results, such a development strand covers academic research and results from laboratories as well as applications. More interestingly, such a new trend in biomimetics could lift the field to a higher level of abstraction and analogy to a more systemic approach (shown by the publications classified as reviews, case studies, tools and concepts). As mentioned before, sustainability has various dimensions and, in the context of biomimetics, it is usually understood as environmentally friendly production so that it has a link to technical innovation. However, the other dimensions can be considered as well or can become the focus of the research, e.g., with social or economic components of sustainability. As such, biomimetics can cover a broad field of sustainability research, and the SDGs offer a framework to specify developments. Additionally, a key finding is that biological systems can serve as models for biomimetic sustainable developments on a molecular level, which might shift biomimetics to technologies that truly implement the abstracted solutions that biological models offer rather than optimizing the traditional way of problem-solving.

## Figures and Tables

**Table 1:** Types of publications. Types of publications and how many of them were found (*n* = 1142). A total of 15 different types of publications could be identified. Most of the publications concerned the presentation of test results in the laboratory (*n* = 601) or topic overviews or summaries (*n* = 427).

Type of Publication	Number (Total *n* = 1142)
Results from laboratory	601
Review	427
Case study	35
Algorithm, tools	24
Application	17
Concept	10
Prototype	9
Study module	5
Biography	1
Conference	1
Interview	1
Process optimization	1
Questionnaire survey	1
Other/insufficient information	9

**Table 2:** List of the top 10 journals publishing on biomimetics and sustainable applications ranked by the number of relevant articles published in each. Only the journals with more than nine papers are shown as the rest of the papers (*n* = 723) were published in 424 different journals with an average of 1.7 papers per journal over the period 2004–2021. ACS: American Chemical Society; RSC: Royal Society of Chemistry.

Journal Name	Total (*n* = 875)	Impact Factor (2021)
ACS Sustainable Chemistry and Engineering	29	9.2
ACS Applied Materials and Interfaces	21	10.4
RSC Journal of Materials Chemistry	18	7.6, 14.5
Elsevier Chemical Engineering Journal	15	16.7
ACS Nano	13	18.0
Wiley Advanced Functional Materials	12	19.9
Wiley Angewandte Chemie—International Edition	12	16.8
MDPI Sustainability	12	3.9
Wiley Chemistry-Sustainability-Energy-Materials	11	9.1
Nature Scientific Reports	9	4.9
All others	723	N/A

**Table 3:** List of the 25 most represented research areas of (A) “biomimetics” and (B) “biomimetics and sustainab*” publications. Retrieved on 16 June 2023 through Web of Science, all databases (1945–2023). Similar areas are marked in green with specific ones marked in blue (A) and yellow (B).

(A) Biomimetics		(B) Biomimetics and Sustainab*	
Research Area (Total *n* = 23.735)	Records	Research Area (Total *n* = 443)	Records
Engineering	15.401	Engineering	280
Material Science	14.317	Materials Science	269
Chemistry	11.777	Science Technology Other Topics	221
Science Technology Other Topics	11.606	Chemistry	200
Physics	10.591	Physics	192
Biotechnology Applied Microbiology	9.438	Biotechnology Applied Microbiology	135
Biochemistry Molecular Biology	8.536	Environmental Sciences Ecology	109
Cell Biology	8.080	Biochemistry Molecular Biology	105
Biophysics	7.458	Energy Fuels	79
Radiology Nuclear Medicine Medical Imaging	4.053	Business Economics	78
Polymer Sciences	3.871	Biophysics	73
Pharmacology, Pharmacy	3.583	Polymer Science	70
Instruments, Instrumentation	3.556	Cell Biology	58
Computer Science	3.514	Education Educational Research	34
Robotics	2.714	Mechanics	33
Physiology	2.693	Pharmacology Pharmacy	33
Mathematics	2.502	Instruments Instrumentation	32
Automation Control Systems	2.113	Crystallography	31
Crystallography	2.096	Electrochemistry	31
Anatomy Morphology	1.936	Radiology Nuclear Medicine Medical Imaging	29
Neuroscience, Neurology	1.933	Plant Sciences	28
Mathematical Computational Biology	1.900	Agriculture	25
Mechanics	1.863	Computer Science	24
Zoology	1.632	Construction Building Technology	21
Life Sciences, Biomedicine, Other Topics	1.528	Robotics	20

**Table 4:** Research areas for the top 50 most cited (A) “biomimetics” and (B) “biomimetics and sustainab*” publications. Retrieved on 16 June 2023 through Web of Science, all databases (1945–2023). For each field, the first ten areas are given with the respective records.

(A) Biomimetics Top 50 Most Cited		(B) Biomimetics and Sustainab* Top 50 Most Cited	
Research Area	Records	Research Area	Records
Science Technology Other Topics	22	Chemistry Multidisciplinary	21
Chemistry	17	Materials Science Multidisciplinary	20
Materials Science	16	Chemistry Physical	16
Engineering	9	Nanoscience Nanotechnology	13
Biochemistry Molecular Biology	6	Physics Applied	11
Physics	6	Physics Condensed Matter	11
Biotechnology Appl. Microbiol.	5	Materials Science Biomaterials	6
Computer Science	5	Energy Fuels	5
Cell Biology	3	Multidisciplinary Sciences	5
Genetics Heredity	2	Engineering Biomedical	4

## Data Availability

Data are available upon request with a commitment for equitable collaboration with the authors.

## Supplementary Materials

The following supporting information can be downloaded at: https://www.mdpi.com/article/10.3390/biomimetics8030304/s1, Table S1: Top 20 most cited papers for “biomimetics” retrieved on 16 June 2023 through Web of Science, all databases; Table S2: Top 20 most cited papers for “biomimetics and sustainab*” retrieved on 16 June 2023 through Web of Science, all databases.
