# Supplementary material for: Biomimetics for Sustainable Developments—A Literature Overview of Trends
Source: Biomimetics (Basel). 2023 Jul 11;8(3):304. doi: 10.3390/biomimetics8030304 (PMC10807477; doi:10.3390/biomimetics8030304)
Supplement: Supplementary file 1 [file biomimetics-08-00304-s001.zip › biomimetics-2429337-supplementary.pdf]

Table S1: Top 20 most cited papers for "biomimetics". Retrieved on June 16, 2023, through Web of Science, all databases.

	Authors	Year	Title	Journal	Document Type	Times cited
1	Armand & Tarascon	2008	Building better batteries	Nature	Article	15438
2	Kokubo & Takadama	2006	How useful is SBF in predicting in vivo bone bioactivity?	Biomaterials	Article	6855
3	Gao et al.	2007	Intrinsic peroxidase-like activity of ferromagnetic nanoparticles	Nature Nanotechnology	Article	4330
4	Rus & Tolley	2015	Design, fabrication and control of soft robots	Nature	Review	3213
5	Zhang	2003	Fabrication of novel biomaterials through molecular self-assembly.	Nature Biotechnology	Article	2782
6	Sheikh & Bovik	2006	Image information and visual quality	IEEE Transactions On Image Processing	Article	2611
7	Tuteja et al.	2007	Designing superoleophobic surfaces	Science	Article	2483
8	Prezioso et al.	2015	Training andoperation of an integrated neuromorphic network based on metal-oxide memristors	Nature	Article	1874
9	Gladman et al.	2016	Biomimetic 4D printing	Nature Materials	Article	1838
10	Zhou et al.	2014	All-Solid-State Z-Scheme Photocatalytic Systems	Advanced Materials	Article	1832
11	Lee et al.	2007	A reversible wet/dry adhesive inspired by mussels and geckos	Nature	Article	1627
12	Kokubo et al.	2003	Novel bioactive materials with different mechanical properties.	Biomaterials	Article	1623
13	Majumder et al.	2005	Nanoscale hydrodynamics - Enhanced flow in carbon nanotubes	Nature	Editorial Material	1567
14	Lee et al.	2011	A review of reverse osmosis membrane materials for desalination-Development to date and future potential	Journal Of Membrane Science	Review	1566
15	Chortos et al.	2016	Pursuing prosthetic electronic skin	Nature Materials	Review	1541
16	Amini et al.	2012	Bone tissue engineering: recent advances and challenges.	Critical Reviews In Biomedical Engineering	Article	1490

17	Hu et al.	2011	Erythrocyte membrane-camouflaged polymeric nanoparticles as a biomimetic delivery platform	Proceedings Of The National Academy Of Sciences Of The United States Of America	Article	1487
18	Shepherd et al.	2011	Multigait soft robot	Proceedings Of The National Academy Of Sciences Of The United States Of America	Article	1473
19	Svenson & Tomalia	2005	Commentary - Dendrimers in biomedical applications - reflections on the field	Advanced Drug Delivery Reviews	Editorial Material	1469
20	Bhushan & Jung	2011	Natural and biomimetic artificial surfaces for superhydrophobicity, self-cleaning, low adhesion, and drag reduction	Progress In Materials Science	Review	1412

Table S2: Top 20 most cited papers for "biomimetics and sustainab*". Retrieved on June 16, 2023, through Web of Science, all databases.

	Authors	Year	Article Title	Journal	Document Type	Times cited
1	Li et al.	2019	Cocatalysts for Selective Photoreduction of CO2 into Solar Fuels	Chemical Reviews	Review	1228
2	Ozbolat & Hospodiuk	2016	Current advances and future perspectives in extrusion-based bioprinting	Biomaterials	Review	857
3	Zhao et al.	2014	Porous Metal-Organic Frameworks for Heterogeneous Biomimetic Catalysis	Accounts Of Chemical Research	Review	619
4	Walther et al.	2010	Large-Area, Lightweight and Thick Biomimetic Composites with Superior Material Properties via Fast, Economic, and Green Pathways	Nano Letters	Article	394
5	Magnuson et al.	2009	Biomimetic and Microbial Approaches to Solar Fuel Generation	Accounts Of Chemical Research	Review	364
6	Wakerley et al.	2019	Bio-inspired hydrophobicity promotes CO2 reduction on a Cu surface	Nature Materials	Article	333
7	Wu et al.	2020	*H2*S-activatable near-infrared afterglow luminescent probes for sensitive molecular imaging in vivo	Nature Communications	Article	290
8	Madeo et al.	2019	Caloric Restriction Mimetics against Age-Associated Disease: Targets, Mechanisms, and Therapeutic Potential	Cell Metabolism	Review	287
9	Zhang et al.	2014	A Biomimetic Copper Water Oxidation Catalyst with Low Overpotential	Journal Of The American Chemical Society	Article	281
10	Solga et al.	2007	The dream of staying clean: Lotus and biomimetic surfaces	Bioinspiration & Biomimetics	Article	244
11	MacVittie et al.	2013	From cyborg lobsters to a pacemaker powered by implantable biofuel cells	Energy & Environmental Science	Article	235
12	Guo et al.	2018	Light-driven fine chemical production in yeast biohybrids	Science	Article	201
13	Lee et al.	2018	Photosynthetic artificial organelles sustain and control ATP-dependent reactions in a protocellular system	Nature Biotechnology	Article	190
14	Kluwer et al.	2009	Self-assembled biomimetic [2Fe2S]-hydrogenase-based photocatalyst for molecular hydrogen evolution	Proceedings Of The National Academy Of Sciences Of The United States Of America	Article	189
15	Li et al.	2018	Recent Advances in Intrinsic Self-Healing Cementitious Materials	Advanced Materials	Article	176
16	Liu et al.	2016	Bioinspired Bifunctional Membrane for Efficient Clean Water Generation	Acs Applied Materials & Interfaces	Article	175
17	Diesendruck et al.	2014	Mechanically triggered heterolytic unzipping of a low-ceiling-temperature polymer	Nature Chemistry	Article	175

18	Zhang et al.	2020	Self-healing cement concrete composites for resilient infrastructures: A review	Composites Part B-Engineering	Review	168
19	Zhao et al.	2014	Biomimetic and bioinspired membranes: Preparation and application	Progress In Polymer Science	Review	163
20	Ghasemlou et al.	2019	Bio-inspired sustainable and durable superhydrophobic materials: from nature to market	Journal Of Materials Chemistry A	Review	146
